# Naphthalene biodegradation under oxygen‐limiting conditions: community dynamics and the relevance of biofilm‐forming capacity

**DOI:** 10.1111/1751-7915.12842

**Published:** 2017-08-25

**Authors:** Sophie‐Marie Martirani‐Von Abercron, Patricia Marín, Marta Solsona‐Ferraz, Mayra‐Alejandra Castañeda‐Cataña, Silvia Marqués

**Affiliations:** ^1^ Estación Experimental del Zaidín Department of Environmental Protection Consejo Superior de Investigaciones Científicas Granada Spain

## Abstract

Toxic polycyclic aromatic hydrocarbons (PAHs) are frequently released into the environment from anthropogenic sources. PAH remediation strategies focus on biological processes mediated by bacteria. The availability of oxygen in polluted environments is often limited or absent, and only bacteria able to thrive in these conditions can be considered for bioremediation strategies. To identify bacterial strains able to degrade PAHs under oxygen‐limiting conditions, we set up enrichment cultures from samples of an oil‐polluted aquifer, using either anoxic or microaerophilic condition and with PAHs as the sole carbon source. Despite the presence of a significant community of nitrate‐reducing bacteria, the initial community, which was dominated by *Betaproteobacteria*, was incapable of PAH degradation under strict anoxic conditions, although a clear shift in the structure of the community towards an increase in the *Alphaproteobacteria* (*Sphingomonadaceae*), *Actinobacteria* and an uncultured group of *Acidobacteria* was observed in the enrichments. In contrast, growth under microaerophilic conditions with naphthalene as the carbon source evidenced the development of a biofilm structure around the naphthalene crystal. The enrichment process selected two co‐dominant groups which finally reached 97% of the bacterial communities: *Variovorax* spp. (54%, *Betaproteobacteria*) and *Starkeya* spp. (43%, *Xanthobacteraceae*). The two dominant populations were able to grow with naphthalene, although only *Starkeya* was able to reproduce the biofilm structure around the naphthalene crystal. The pathway for naphthalene degradation was identified, which included as essential steps dioxygenases with high affinity for oxygen, showing 99% identity with *Xanthobacter polyaromaticivorans dbd* cluster for PAH degradation. Our results suggest that the biofilm formation capacity of *Starkeya* provided a structure to allocate its cells at an appropriate distance from the toxic carbon source.

## Introduction

Polycyclic aromatic hydrocarbons (PAHs) are toxic compounds of particular environmental concern because of their marked stability and resistance to degradation. They are released into the environment either from natural sources or as a result of industrial activities. The simplest PAH naphthalene is included among the 16 PAHs considered to be priority pollutants by the US EPA. International efforts to improve remediation strategies for hydrocarbon‐polluted sites focus on biological processes mediated by bacteria. Bacteria have developed a striking adaptive capacity for degrading natural and synthetic aromatics to CO_2_ and water, which is the basis for the use of bioremediation approaches to clean aromatic‐polluted areas.

Bacteria able to degrade PAHs under strict aerobic conditions are widespread and have been widely described. The biodegradation pathway involves the deeply characterized aromatic mono‐ and dioxygenases for ring activation (hydroxylation) and ring cleavage (Vaillancourt *et al*., 2006; Ullrich and Hofrichter, 2007). The genes and gene organization of PAH degradation operons are reasonably conserved among PAH‐degrading isolates, especially among the *Pseudomonads*, which are predominant in the databases of PAH degraders. However, the availability of oxygen is often limited in natural environments because oxygen can be rapidly consumed in the aerobic biodegradation processes, resulting in a decreasing oxygen concentration gradient whereupon reduction of other electron acceptors such as nitrate, iron or sulfate becomes energetically favourable (Rivett *et al*., 2008). A similar pattern is observed in habitats that are under permanent anoxic conditions, such as flooded sediments in marine and freshwater environments (Brune *et al*., 2000). Because nitrate respiration is not a strictly anoxic process, aerobic and nitrate‐reducing metabolisms generally coexist at the boundaries of polluted sites, where microaerophilic conditions are generally found (Wilson and Bouwer, 1997).

Under strict anoxic conditions, PAH biodegradation has only been reliably proved in sulfate‐reducing bacteria (SRB), although only one *Deltaproteobacteria* strain and a two‐strain consortium able to degrade naphthalene have been isolated and characterized to date (Meckenstock *et al*., 2016). Anaerobes use a reductive strategy to overcome the stability of the aromatic ring. Naphthalene must be converted to 2‐naphthoic acid (NA) (Zhang and Young, 1997) previous to its activation to naphthoyl–CoA, which is then de‐aromatized, starting from the non‐activated ring, by the activity of two specific reductases: a naphthoyl–CoA reductase (*ncr* gene) followed by a 5,6‐dihydro‐2‐naphthoyl–CoA reductase (DHNCR; Eberlein *et al*., 2013a; Estelmann *et al*., 2015; ). The subsequent dearomatization of the activated ring requires the activity of a 5,6,7,8‐tetrahydro‐2‐naphthoic acid reductase (THNCR; Eberlein *et al*., 2013b). Degradation of 2‐methylnaphthalene (2MN) requires the addition of its methyl group to fumarate through a naphthyl‐2‐methyl succinate synthase (*nmsABC* genes), followed by β‐oxidation‐like steps to finally render naphthoyl‐CoA (Meckenstock *et al*., 2016). The degradation of PAHs under nitrate‐reducing conditions has been detected in the environment, and the presence of nitrate‐reducing bacteria (NRB) communities able to degrade PAHs in environmental samples has been quantified in a number of sites (Eriksson *et al*., 2003; Uribe‐Jongbloed and Bishop, 2007; Acosta‐González *et al*., 2013; Martirani‐Von Abercron *et al*., 2016). However, to date, no nitrate‐reducing bacteria (NRB) isolate able to reproducibly degrade PAHs under strict anoxic conditions have been reported. Recently, the description of intracellular oxygen production from nitrate respiration through a putative nitric oxide dismutase (Zedelius *et al*., 2011; Ettwig *et al*., 2012) and the discovery of a high abundance of the corresponding *nod* gene in the environment (Zhu *et al*., 2017a) have opened up the possibility of the utilization of aerobic degradation pathways under anoxic, denitrifying conditions. However, the actual functioning of this supposed oxygen‐producing pathway under real conditions for the degradation of hydrocarbons and other pollutants has not been proven. Furthermore, most nitrate‐reducing bacteria are facultative anaerobes that may alternate nitrate and oxygen respiration according to the availability of terminal electron acceptors, which is likely to fluctuate in polluted environments. Under microaerophilic prevailing conditions, minute amounts of oxygen suffice to promote the aerobic degradation of hydrocarbons (Yerushalmi *et al*., 2002; Táncsics *et al*., 2013).

Experimental approaches in the laboratory have mainly focussed on PAH degradation by organisms in the planktonic state. However, in the environment, most microbial processes occur through the development of multispecies biofilm where several groups of bacteria coexist and interact. Natural biofilms are complex supracellular structures composed of different species that are self‐organized and coordinated to generate an appropriate habitat for the community (Flemming *et al*., 2016). Growth in biofilm structures provides a number of advantages for the members of the consortium as compared to growth in the planktonic state (Roder *et al*., 2016). Fundamental to these biofilm properties is the ability to produce extracellular polymeric substances (EPS), which support the architecture of the biofilm, and provides mechanical stability, physical proximity, protection against stressors and predators, water retention and passive sorption of solutes, among other benefits. Furthermore, interaction between species, which are favoured in complex biofilms, may lead to the development of new capabilities resulting either from the joint activity of members of the consortium (i.e. synergy (Ren *et al*., 2015), or from the transfer and combination of functions in a single strain (i.e. horizontal gene transfer (Madsen *et al*., 2012; Roberts and Kreth, 2014)).

Here, we investigate the fate of microbial populations exposed to PAHs under conditions resembling natural situations. To this end, we started from samples of a hydrocarbon‐polluted aquifer where a natural bacterial biofilm had developed at the oil–water interface, and we used parallel approaches under different oxygen‐limiting conditions that bacteria may face in the environment: complete anoxia with nitrate as electron acceptor, which are found when the available oxygen is completely consumed and there is no replenishment, and microaerophilic conditions, which are those generally found at the boundaries of contaminated plumes. We followed changes in the microbial communities during enrichment with PAHs in static set‐ups. In these conditions, we expected active enrichment of those strains adapted to thrive more efficiently under a respiratory regime that resembles the conditions found in natural‐polluted sites using the provided PAH as the carbon source.

## Results and discussion

### *Betaproteobacteria* are dominant in a hydrocarbonpolluted aquifer

In the initial sample collected from a 25‐m‐deep well of a hydrocarbon‐polluted aquifer, a biofilm spreading between the water phase and the upper 10‐cm oil layer was clearly visible (Fig. S1). The most abundant PAHs in the water phase were two‐ring aromatics (0.14 ± 0.082 ppm of naphthalene, 0.20 ± 0.119 ppm of 2‐methylnaphthalene) followed by one order of magnitude lower levels of aromatics with three or more rings (Table S1). The alkane distribution was coherent with contamination with a mixture of diesel and gasoline (Fig. S2). The concentration of nitrate and sulfate in the aqueous phase was in the range of natural background levels in oligotrophic groundwaters (Einsiedl and Mayer, 2005; Arauzo and Martínez‐Bastida, 2015; Table S2).

**Figure 1:**
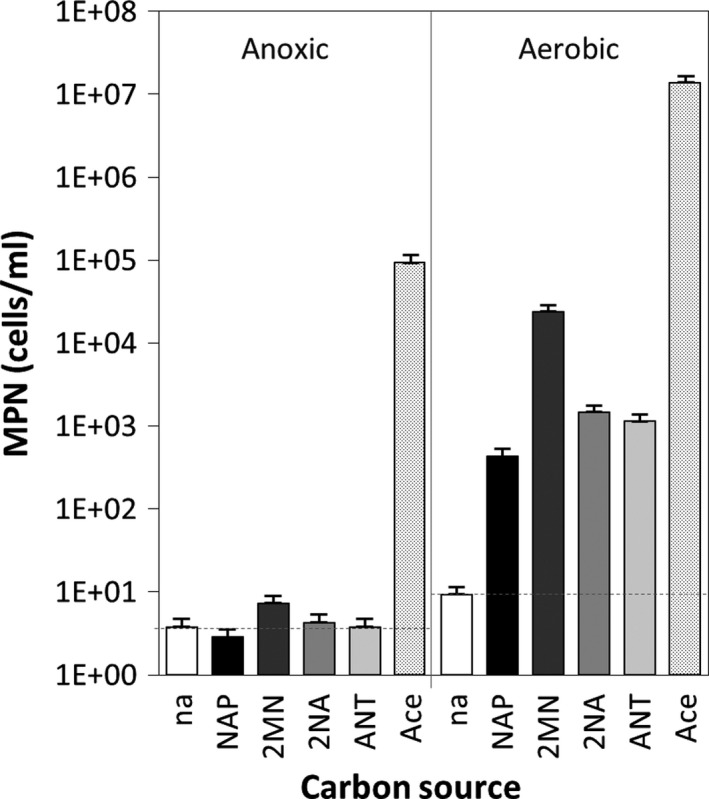
Most probable number enumeration in the aquifer initial sample of nitrate‐reducing and aerobic bacteria able to grow on naphthalene (NAP), 2‐methylnaphthalene (2MN), 2‐naphthoic acid (2NA), anthracene (ANT), acetate (Ace) and with no added carbon source (na). Counts were made in triplicate. Numerical values (95% CI) can be found in Table S1. Dotted lines indicate the basal growth with no added carbon source.

The potential of the initial biofilm community to degrade PAHs was determined using the most probable number (MPN) enumeration of aerobic and nitrate‐reducing bacteria. Naphthalene (NAP), 2MN, 2‐naphthoic acid (2NA, an intermediate in anaerobic naphthalene degradation) and anthracene (ANT) were used as carbon sources. A positive control with acetate as the carbon source and a negative control with no addition were included in the analysis to estimate the size of the nitrate‐reducing community and the basal growth on the sample's intrinsic organic matter respectively. Under anoxic conditions, the high counts (10^5^ cells ml^−1^) of NRB able to grow with acetate revealed the presence of a significant community of NRB in the biofilm (Fig. 1, Table S3). However, no growth above the basal level was observed with any of the aromatics used, except for a minor increase with 2MN, indicative of the absence of NRB able to use naphthalenes or anthracene. This contrasted with the results obtained under aerobic conditions; substantial growth on the three aromatics was observed, which ranged between 4.3 × 10^2^ cells ml^−1^ of naphthalene degraders and 2.4 × 10^4^ cells ml^−1^ of 2MN degraders, consistent with the higher levels of 2MN in the polluted aquifer (Table S1). These values represented only a minor fraction of the culturable aerobic community present, which reached 1.4 × 10^7^ cells ml^−1^ when acetate was used as the carbon source.

**Figure 2:**
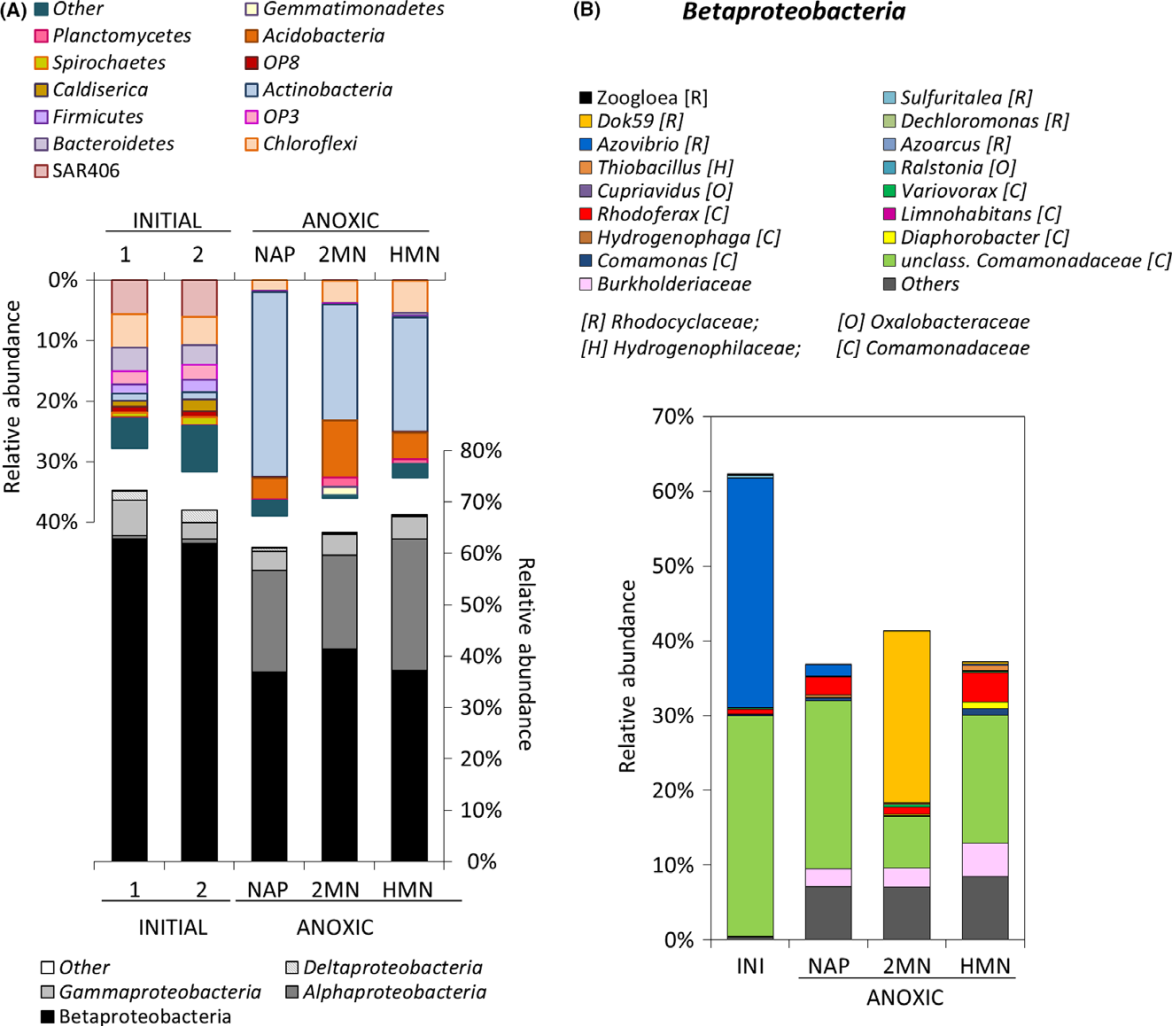
A. Cumulative plot of bacterial phyla detected in the initial environmental samples and in the anaerobic enrichments amended with naphthalene (NAP), 2‐methylnaphthalene (2MN) and heptamethylnonane (HMN). The *Proteobacteria* are described at the class level. The initial samples were analysed in duplicate (labelled 1 and 2). B. Detailed cumulative plot of the *Betaproteobacteria* detected in the same samples. The average values of the duplicate initial samples shown in A are included for comparison, labelled as INI. *[R], Rhodocyclaceae; [O] Oxalobacteraceae; [H] Hydrogenophilaceae; [C] Comamonadaceae*. Numerical values can be found in Table S4.

We isolated the total DNA of duplicate biofilm samples taken at the oil–water interface to determine the structure of the community present using 16S rRNA gene V1–V3 region pyrosequencing. Total read numbers generated from all the samples analysed in this study and the corresponding diversity indices are summarized in Table 1. The rarefaction curves (Fig. S3) and the Good's sample coverage estimator suggested that sampling depth was sufficient in all cases to estimate the microbial diversity (Table 1). The observed OTU number, diversity indices and OTU distribution of the initial sample were reproducible between replicas. Figure 2 shows that the biofilm community was clearly dominated by the *Proteobacteria*, the *Betaproteobacteria* accounting for more than 60% of the bacterial population. *Bacteroidetes*,* Chloroflexi* and the uncultured lineage SAR406 (also referred to as Marine Group A (Wright *et al*., 2014)) were also present in substantial levels. The major presence of *Betaproteobacteria*,* Bacteroidetes* and *Chloroflexi* in this hydrocarbon‐polluted site is in agreement with the strains frequently found and isolated from aquifers and groundwater environments (Griebler and Lueders, 2009), especially when carbon source availability is high (Fierer *et al*., 2007). The *Betaproteobacteria* community in the sample was relatively simple, dominated by two operational taxonomic units (OTUs): one belonging to the *Comamonadaceae* (29.5%) and a *Rhodocyclaceae* closely related to *Azovibrio* (30.6%; Fig. 2B). *Betaproteobacteria* belonging to the *Comamonadaceae* occur in diverse pristine and polluted habitats such as soil, freshwater, groundwater and industrial processing water and can use a variety of substrates, including hydrocarbons, as carbon source (Willems, 2014). Interestingly, members of this group were also prevalent in toluene‐polluted model wetlands and in BTEX‐contaminated groundwater (Táncsics *et al*., 2013; Martinez‐Lavanchy *et al*., 2015). The *Gammaproteobacteria* were almost exclusively composed of a single OTU closely related to *Pseudoxanthomonas* (3.8%) (Table S4).

**Table 1:** Comparison of OTU number, diversity, evenness indices and coverage for the different samples

Sample^a^	NS^b^	OTUs^c^	OTUs (1500)^d^	Chao1 (1500)^e^	Shannon	Coverage^f^
INI_a_	1807	167	158	259.08	4.08	95.87%
INI_b_	1762	174	168	248.50	4.29	96.19%
Anox‐NAP	3611	138	101	207.25	4.24	98.22%
Anox‐2MN	6778	161	84	189.86	4.48	99.13%
Anox‐HMN	3992	148	104	198.23	4.57	98.57%
Micro5‐N	34 611	115	42	61.13	2.66	99.94%
Micro15‐N	22 599	69	26	65.00	1.35	99.91%
Micro‐12s‐N	23 347	101	43	58.00	2.33	99.91%
Aer15‐N	29 889	111	37	64.20	1.58	99.89%
Aer19‐N	20 685	116	45	129.33	2.46	99.79%

a INI_a_ and INI_b_ refer to the two replica of the initial samples.

b Number of sequences for each library filtered for chimera and singletons.

c OTU numbers calculated with all sequences at the 3% distance level.

d OTU numbers calculated for a randomized subset of 1500 reads per sample at the 3% distance level.

e Chao index calculated with 1500 subsampled sequences.

f Good's sample coverage estimator.

Using samples from this biofilm layer, we set up a series of enrichment cultures under two different oxygen limitation regimes to select PAH‐degrading communities.

### Changes in the community structure under strictly anoxic denitrifying conditions

Enrichment cultures with nitrate as the electron acceptor and naphthalene and 2MN as the only carbon source were prepared under strict anoxic conditions (see the *Experimental procedures* section). To reduce the PAH concentration in the water phase, which might limit growth of anaerobes (Galushko *et al*., 1999), the PAHs were provided dissolved in 2,2,4,4,6,8,8‐heptamethylnonane (HMN). After 12 months and three subsequent subcultures, DNA from the anoxic cultures was isolated and analysed as above by 16S rRNA 454 pyrosequencing to determine changes in the structure of the community (samples Anox‐NAP, Anox‐2MN, Anox‐HMN; Table 1). A moderate loss of diversity was observed in all the nitrate‐reducing cultures, together with a clear shift in the community structure that may be attributed to the imposed change in the respiratory regime. Overall, the changes were similar in the presence of the PAHs and when only the carrier HMN was added to the cultures (Fig. 2A, Table S4). Whilst the dominant *Commamonadaceae* detected in the starting material was still present at high levels in the NAP, HMN and to a lesser extent in the 2MN cultures, the dominant *Azovibrio* OTU disappeared almost completely in the three enrichments, partially balanced by an increase in *Betaproteobacteria* representatives belonging to *Burkholderiaceae*,* Rhodoferax* and especially to the *Rhodocyclaceae* genus Dok59 in the case of the 2MN cultures (Fig. 2B). Members of the *Bacteroidetes*, which constituted more than 3% of the initial community, decreased to levels below 0.2% in all conditions. The strong decrease of some groups in all the enrichments suggests their high sensitivity to the new culture conditions, that is, strict anoxic nitrate respiration and presence of PAHs. In contrast, an increase in *Alphaproteobacteria* was observed in all conditions, especially represented by *Sphingomonadaceae*,* Acetobacteraceae* and *Rhizobiaceae* (Table S4). This might be explained by the denitrifying capacity of some members of the *Sphinomonadaceae* and *Rhizobiaceae*, in addition to their ability to tolerate the presence of toxic hydrocarbons (Carareto Alves *et al*., 2014; Cua and Stein, 2014). The *Anaerolineae* within the *Chloroflexi* also increased significantly in the 2MN and HMN enrichments. As members of this class seem incapable of nitrate respiration, enrichment of this group is probably a consequence of their capacity for fermentative metabolism on cell remains (Yamada and Sekiguchi, 2009). Some specific changes were observed that could be attributed to the presence of PAHs: the *Actinobacteria*, which were almost undetectable in the initial biofilm sample, increased up to 18% in the HMN and 2MN cultures and to almost 30% of the community in the NAP enrichment. Although an increase in the abundance of *Actinobacteria* has been related to the degradation of PAHs in aerobic conditions (Muangchinda *et al*., 2017; Zhu *et al*., 2017b), there is no evidence of the contribution of this phylum to anaerobic degradation. Interestingly, an increase in the *Acidobacteria* candidate class iii1‐8 (order DS‐18) from undetectable levels in the initial biofilm sample to more than 9% of the community in the 2MN enrichment was also observed (Fig. 2A, Table S4), in agreement with a previous observation of a dramatic increase of this poorly characterized group under nitrate‐reducing conditions, and especially in the presence of PAHs (Martirani‐Von Abercron *et al*., 2016). This supports the suggestion that the group is composed of slow‐growing oligotrophs (Fierer *et al*., 2007), probably highly resistant to PAHs (Martirani‐Von Abercron *et al*., 2016). During the enrichment process under anoxic conditions, the minor changes in PAH concentration were not statistically significant (not shown), which reflects the absence of anaerobic PAH degraders determined with the MPN enumeration. Furthermore, our attempts to detect genes involved in anaerobic PAH degradation using primers against *ncr* and *nmsA* in the initial sample and in the enrichment cultures gave negative results. Neither was any amplification product obtained with primers against nitric oxide dismutase gene (*nod*). To date, no growth of NRB isolates with PAHs as the carbon source has been observed under strict anoxic conditions, and in most cases, laboratory conditions can only reproduce aerobic degradation (Yagi *et al*., 2010). The observed shift in the bacterial community describes the adaptation to the new respiratory regime. However, NRBs are facultative anaerobes prepared to thrive in the oxic‐anoxic transition zone, and in these environments, anaerobic respiration and PAH degradation (aerobic) may be spatially separated concomitant processes.

### Microaerophilic conditions strongly select for two dominant strains

**Figure 3:**
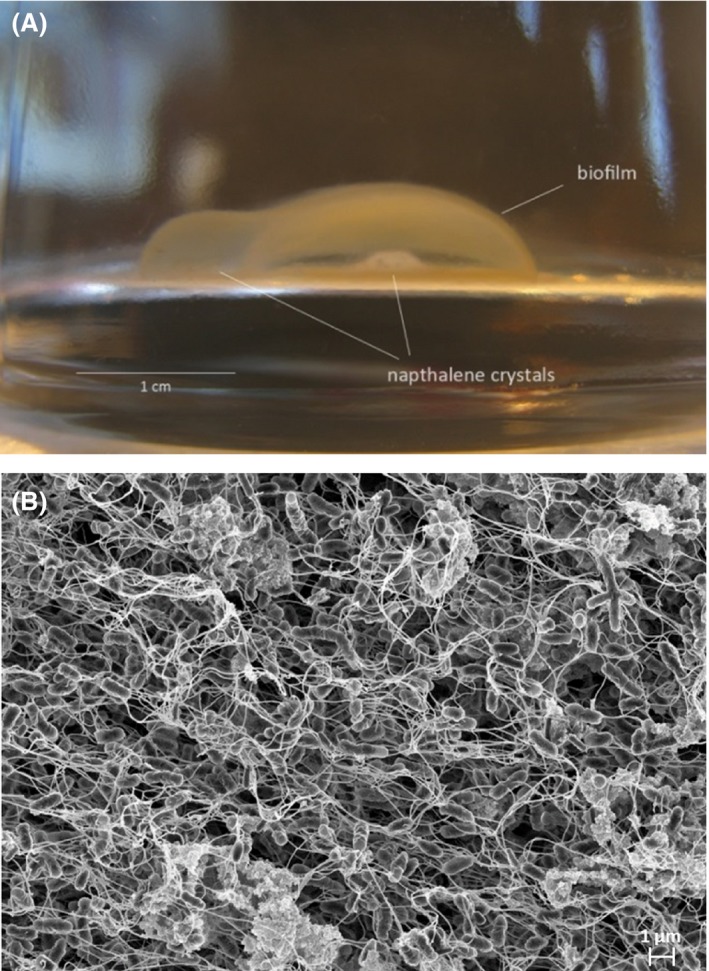
A. Enrichment culture bottle under microaerophilic conditions showing the characteristic bacterial biofilm developed around the naphthalene crystal. B. Scanning electron microscopy (SEM) micrograph showing details of the multispecies biofilm formed around the naphthalene crystal during growth under microaerophilic conditions. Scale bar = 1 μm.

**Figure 4:**
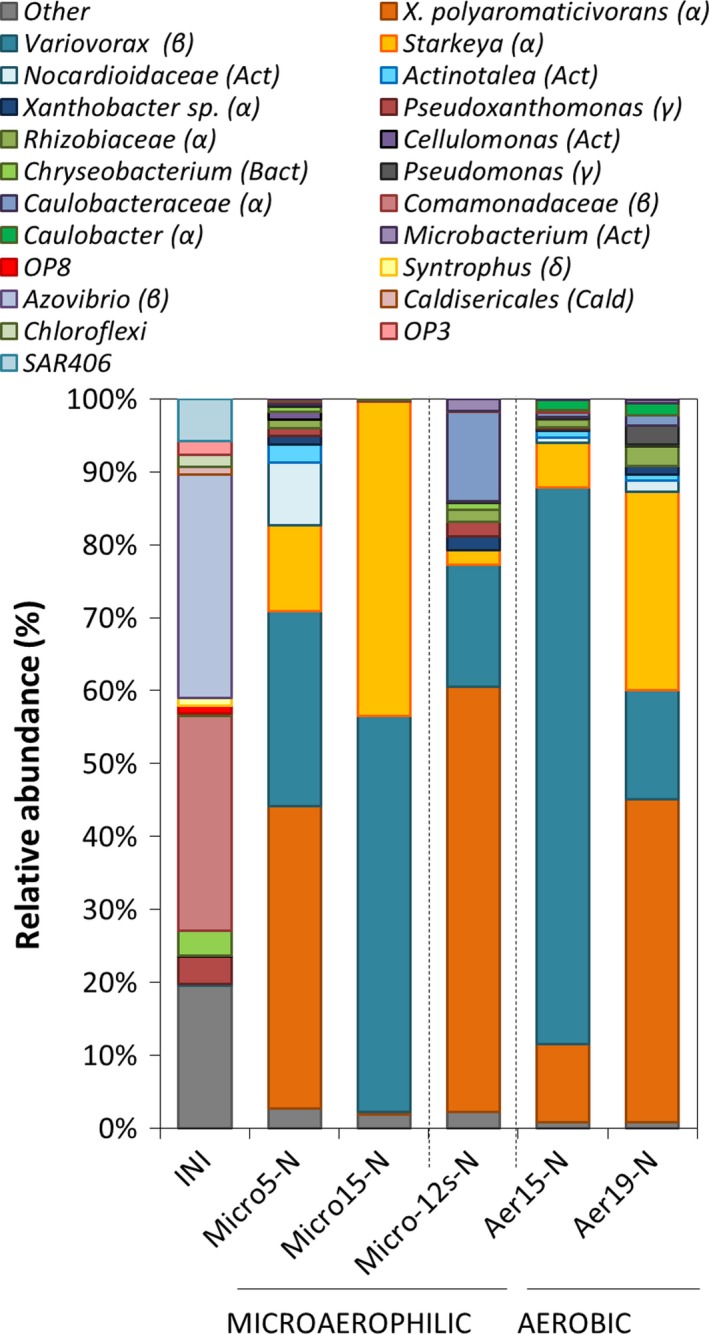
Cumulative plot of bacterial taxons present at more than 1% of the community detected in the microaerophilic, aerobic and synthetic microaerophilic enrichments amended with crystals of naphthalene as carbon source. The deepest taxonomic affiliation reached (phyla indicated in brackets) is shown. The average values of the duplicate initial samples (Fig. 2A) are included for comparison, labelled as INI. Numerical values can be found in Table S4.

To mimic the natural habitat of most NRB, which are facultative anaerobes that can thrive at low oxygen concentrations, we set up microaerophilic enrichment cultures. To this end, culture bottles were initially flushed with nitrogen gas to reach anoxic conditions; the caps of the bottles were prepared to allow a slow influx of air (see *Experimental procedures* section) so that in the absence of inoculum oxygen saturation in the medium could be reached after approximately 1 week (Fig. S4B). In this set‐up, inoculation of the medium resulted in oxygen consumption, which increased gradually to reach rates at equilibrium with the oxygen influx rate. Further growth of the culture caused oxygen consumption at higher rates than diffusion, leading to a progressive decay in the oxygen saturation level, which finally reached undetectable values in spite of the constant oxygen influx. We initiated an enrichment culture using naphthalene as the representative PAH, provided as the sole carbon source in the form of crystals. A fraction of 10% of the culture was regularly transferred to fresh medium. After 3 months, a fully developed biofilm was clearly visible at 0.5–1 cm around the naphthalene crystal in all the cultures in the enrichment process (Fig. 3A). The subsequent subcultures were characterized by the formation of a similar biofilm structure at a short distance from the naphthalene crystal. The crystal was progressively consumed until it completely disappeared. Oxygen concentration at the biofilm level remained close to zero once the biofilm had developed, as previously shown for single species bacterial biofilms (Staal *et al*., 2011). Scanning electron microscopy (SEM) of the developed biofilm revealed the presence of a mixed community of bacteria with different sizes and shapes, adsorbed or attached onto filamentous, sometimes amorphous, EPS material (Fig. 3B). From subcultures 5 (8 months, 5 transfers) and 15 (26 months, 15 transfers), total DNA was isolated and the structure of the community in the enrichments was analysed as above (samples Micro5‐N and Micro15‐N, Table 1). Chao indices and rarefaction curves indicated that during microaerophilic enrichment, the diversity of the bacterial community was gradually reduced, reaching the lowest values in subculture 15 (Fig. S3). Accordingly, a progressive selection for specific groups was observed in the community: the enrichment process initially selected for four dominant *Proteobacteria* in subculture 5, which in some cases could be affiliated to the genus level after comparing the pyrosequencing reads with the 16S rRNA sequence of strains isolated from the enrichments (see below; Fig. 4). The four selected taxa belong to *Xanthobacteraceae* (*Xanthobacter* and *Starkeya*), *Commamonadaceae* (*Variovorax*) and *Nocardiaceae*. Further enrichment under these conditions (subculture 15) finally selected for two co‐dominant strains which constituted 97% of the community: *Variovorax* (54%) and *Starkeya* (43%) and consequently the slowest Shannon evenness index characterized this community (Table 1). It is worth noting that the two enriched strains were almost undetectable in the initial sample.

From subculture 5, we were able to isolate a number of strains on rich medium and on minimal medium with naphthalene as the carbon source that were further characterized (Table 2; Fig. S5). Taxonomic affiliation of the strains matched those taxa which were among the most abundant in the enrichment, although we were unable to isolate some groups, such as *Actinotalea* and the *Nocardiaceae*. Because pyrosequencing only targeted the V1–V3 fragment of 16S rRNA, several OTUs in the pyrosequencing data were assigned to the taxon ‘*Xanthobacteraceae*’, resulting in an ambiguous linking of the OTUs to the isolates. This ambiguity was solved by constructing a phylogenetic tree based on the V1–V3 region of all *Xanthobacteraceae* present in the pyrosequencing data and of the isolates (Fig. S6). Results assigned the most abundant *Xanthobacteraceae* in the final enrichment (subculture 15) to *Starkeya novella*, which corresponded to strain 964 isolated on solid medium with naphthalene as the sole carbon source, and this was designated as *S. novella* strain N1B. The most abundant strain in the enriched community was affiliated to *Variovorax paradoxus* (Fig. 4, Table S4), which corresponded to strain 968 isolated on rich medium, and was designated as *V. paradoxus* strain N2. Interestingly, the isolate 966, which constituted almost 12% of the subculture 5 community, disappeared almost completely in the final enrichment. This strain was finally assigned to *Xanthobacter polyaromaticivorans* (99% 16S rRNA gene identity) based on the *Xanthobacteracea* phylogenetic tree, although it was also closely related to *Aquabacter spiritensis* (98%; Fig. S6). The remaining isolates represented strains that were present at levels below 1% in the final Micro15‐N enrichment (Table 2).

**Table 2:** Strains isolated from the microaerophilic enrichment cultures

Clon #	Isolated from Subculture	Closest relatives (Class)^a^	% ident.	Pyroseq QIIME assigned taxon	% in the Subcultures		Growth on NAP^b^	Biofilm^c^	*dbdB*	*nahA*
					Sub 5	Sub 15				
964	5, 15	*Starkeya novella* (B)	99%	Xanthobacteraceae, Other	26.71	43.19	++	++	+	–
966	5	*Xanthobacter polyaromaticivorans* (B)	99%	Xanthobacteraceae, g_	11.77	0.38	+++	–	+	–
960	5	*Rhizobium naphthalenivorans* (A)	99%	Rhizobiaceae, g_	1.13	<0.01	+	–	–	–
951	5	*Brevundimonas mediterranea* (A)	100%	Caulobacteraceae	0.34	<0.01	++	–	–	–
952	5	*Microbacterium paraoxydans* (Act)	99%	Microbacterium	0.04	<0.01	±	–	–	–
983	5	*Microbacterium* sp. (Act)	99%	Microbacterium	<0.01	<0.01	++	–	–	–
958	5, 15	*Variovorax ginsengisoli* (B)	99%	Variovorax	41.46	54.22	–	–	–	–
968	5, 15	*Variovorax paradoxus* (B)	99%	Variovorax			++	–	–	–
962	5	*Pseudoxanthomonas spadix* (G)	99%	Pseudoxanthomonas	1.13	<0.01	+	–	–	–
943	5	*Pseudomonas nitroreducens* (G)	99%	Pseudomonas	0.37?	<0.01	–	–	–	–
963	5	*Pseudomonas veronii* (G)	99%	Pseudomonas			±	–	–	+
972	5	*Chryseobacterium* sp. (Bact)	98%	Flavobacteriaceae, Other	0.07	<0.01	–	–	–	–

a (B), *Betaproteobacteria*; (A), *Alphaproteobacteria*; (Act), *Actinobacteria*; (G); *Gammaproteobacteria*; (Bact), *Bacteroidetes*.

b Determined after 18 days of growth at 28°C and moderate shaking speed (100 rpm). +++ OD > 0.9; ++ OD > 0.4; + OD > 0.3; ± OD < 0.1.

c Biofilm developed around the naphthalene crystal.

### A pathway with high affinity for oxygen is favoured under microaerophilic conditions

Eight strains among the isolates, including the dominant *Variovorax* and *Starkeya* strains, were able to grow aerobically with naphthalene as the carbon source (Table 2). To identify the genes involved in naphthalene degradation, we used previously described primers and newly designed degenerated primers based on the alignment of 29 NahA sequences for naphthalene dioxygenases belonging to different taxonomic groups (Table S5, see the *Experimental procedures* section). PCR analysis with several sets of primers to amplify naphthalene dioxygenase gene was carried out with DNA from the enrichments and from all the isolates. Only *Pseudomonas veronii* and the total DNA from subculture 5 gave positive results with *nahA* primers. The *P. veronii nahA* gene product clustered with genes for class A ring‐hydroxylating dioxygenases and was predicted to utilize alkyl substituted monoaromatics as well as PAHs as substrate, whilst subculture 5 *nahA* gene product was predicted to prefer hetero‐polycyclic hydrocarbons and arylbenzenes, according to the ring‐hydroxylating oxygenase database (Chakraborty *et al*., 2014; Fig. S7). As *X*. *polyaromaticivorans* was present among the isolates and was the predominant strains in the subculture 5 community, we designed and assayed primers for the *dbdB* gene coding for a dioxygenase from *X*. *polyaromaticivorans* strain 127W naphthalene/dibenzothiophene degradation pathway (Hirano *et al*., 2004). Only the isolates belonging to the *Xanthobacteraceae*,* S. novella* and *X. polyaromaticivorans*, produced the corresponding PCR amplification products, which showed 99**%** identity with the *X. polyaromaticivorans dbdB* sequence (Fig. S7). We further investigated the presence of the complete PAH degradation cluster identified in *X. polyaromaticivorans* 127W in *S. novella* strain N1B. We were able to amplify the genes for *dbdABCaCbCcDE* from the *S. novella* strain N1B DNA (Table 3), and this showed a remarkable gene identity (99%) with the described *X. polyaromaticivorans* PAH degradation gene cluster, including a *traA* conjugation gene located upstream *dbdA*. The high percentage of identity of the sequences at the DNA level and the presence of flanking transfer genes suggest that *S. novella* strain N1B could have acquired the naphthalene degradation pathway through a recent horizontal gene transfer event, favoured by the stable physical environment for cell‐to‐cell contact provided by the biofilm matrix. In fact, we were able to detect *X. polyaromaticivorans* in a gene library of whole‐length 16S rRNA gene constructed with DNA from subculture 9 (not shown), and the strain could even be isolated on naphthalene plates from subculture 14. It is worth noting that no plasmid was detected in *S. novella* strain N1B genome. The DbdCa protein, which was shown to code for the alpha subunit of a naphthalene hydroxylating dioxygenase, is closer to group IV dioxygenases utilizing benzene/toluene/biphenyl as substrate, although it showed a preference for larger substrates and was functional at extremely low oxygen concentrations (Hirano *et al*., 2006). The *dbdB* gene codes for a gentisate 1,2‐dioxygenase with high affinity for oxygen (Hirano *et al*., 2007). This suggests that the microaerophilic conditions were selecting for a degradation pathway that included enzymes with high affinity for oxygen. The selection for organisms bearing specific aromatic degradation enzymes observed under microaerophilic conditions, both in competition experiments and in natural environments, has been attributed the high affinity for oxygen of the selected mono‐ and dioxygenases (Balcke *et al*., 2008; Kiesel *et al*., 2008; Táncsics *et al*., 2013; Martinez‐Lavanchy *et al*., 2015). This supports the idea that high oxygen tensions (i.e. ‘absolute’ aerobic conditions) are not essential for aromatic degradation as long as the appropriate organisms are present. RT‐PCR amplification of a *dbdCa* gene fragment from subculture 15 showed that the gene was expressed in these conditions (Fig. S8). The naphthalene degradation genes of *Variovorax* and of the remaining strains, except *Pseudomonas,* could not be identified with the different sets of primers tested. *Variovorax* strains are frequently found in polluted environments and some of them, which bore genes for aromatic ring‐hydroxylating dioxygenases with diverse suggested functions, were capable of aromatic degradation (Satola *et al*., 2013) (Posman *et al*., 2017). However, a specific gene for naphthalene degradation has not been described in this genus. Previous studies have shown that the diversity of the aromatic dioxygenases alpha subunit is underrepresented in the databases, which are dominated by *Pseudomonas*‐related sequences (Gomes *et al*., 2007; Iwai *et al*., 2011), and the use of primers designed to select for substrate specificity does not guarantee the detection of the targeted specific activities (Witzig *et al*., 2006).

**Table 3:** Naphthalene degradation gene sequences identified in *S. novella* strain N1B

Genes	Amplicon size	% Id.	Function
*dbdAB’*	994	98%	Ferredoxin (*dbdA*); gentisate 1,2‐dioxygenase (*dbdB*) (preceded by a partial *traA* gene coding for a conjugative transfer relaxase)
*dbdCa*	1398	99%	Naphthalene 1,2‐dioxygenase alpha subunit
*dbdCbCc*	1556	99%	Naphthalene 1,2‐dioxygenase beta subunit (*dbdCb*) Ferredoxin (*dbdCc*)
*dbdD*	1078	99%	Dihydrodiol dehydrogenase

### Evolution of a synthetic community under microaerophilic conditions

To gain further insight into the community structure dynamics, we reproduced an artificial microbial community by mixing equal proportions of the twelve isolates (Table 2) in a single microaerophilic culture, which was then subjected to a similar selective pressure using naphthalene as the sole carbon source for 12 weeks. The structure of the resulting community was analysed by 16S rRNA pyrosequencing as above (Table 1, sample Micro‐12s‐N). Because the only possible strains present in this culture were the twelve isolates, we used the pyrosequencing data from this community to build the phylogenetic tree of the different taxons to establish the link between QIIME affiliation and strain isolates (Fig. S6 and not shown). Figure 4 (sample Micro‐12s, Table S4) shows that the community had evolved from an initially even situation with equally abundant strains, to a community where the naphthalene degrading strains *X. polyaromaticivorans* and to a lesser extent *Brevundimonas* and *Variovorax* dominated. Some *Brevundimonas* species are characterized for their dependence on microaerophilic conditions for growth (Buczolits *et al*., 2001). In contrast, the abundance of *Microbacterium*,* Starkeya*,* Pseudoxanthomonas* and *Rhizobium*, which were also capable of naphthalene degradation, was reduced to between 1.5% and 2% of the community. Finally, the two *Pseudomonas* strains and *Chryseobacterium* sp., unable to use naphthalene as a carbon source, almost disappeared, in accordance with their low abundance in the enriched subcultures 5 and 15. Overall, the distribution resembled the community structure in subculture 5 and probably represented an intermediate step in the evolution of the community.

### Aerobic growth allows higher bacterial diversity

To analyse the impact of oxygen tension on the evolution of the community, subculture 5 of the microaerophilic enrichment was used as starting material to set‐up enrichment cultures under higher oxygen concentrations provided through continuous shaking at 100 rpm. The oxygen saturation level in the cultures in these conditions was 92%. Samples were taken for community analysis as above after 5 months (10 transfers, sample Aer15‐N) and 9 months (14 transfers, Aer19‐N). Enrichment under aerobic conditions initially favoured *Variovorax* to the detriment of *X. polyaromaticivorans* and further evolved to a structure closer to subculture 5 as regards the dominant OTUs, though with a higher *Starkeya* to *Variovorax* ratio (Fig. 4). In addition, the final sample Aer19‐N also showed a clear increase in overall bacterial diversity (Table 1), where the previously least favoured groups such as *Pseudomonas*,* Rhizobiaceae*,* Caulobacteraceae* and *Microbacterium* recovered to levels well above 1% of the community. Despite the constant shaking of the flaks, the cells showed a strong tendency to form clumps in the medium, which probably creates microhabitats of different oxygen concentrations, which may explain the fluctuations observed in the structure of the community with time.

### *Starkeya novella* strain N1B is responsible for biofilm formation and naphthalene degradation

We analysed the capacity of the different isolates to reproduce the biofilm structure around the naphthalene crystal. Almost all the isolates were capable to certain extent of forming biofilm at the air/liquid interface in rich medium, as determined with the standard crystal violet assay (Fig. S9), both in the presence and in the absence of naphthalene crystals. However, only the *Starkeya novella* strain N1B was able to develop a well‐defined biofilm structure in minimal medium around the naphthalene crystal similar to the one developed by the bacterial community in the microaerophilic enrichments. No growth was observed in these conditions when the naphthalene crystal was omitted from the medium. Interestingly, *X*. *polyaromaticivorans* was among those strains that were less efficient in standard biofilm formation, together with *Pseudoxanthomonas* and *Rhizobium*. In contrast, the two *Variovorax* isolates were characterized by the formation of clumps during growth both in the presence and in the absence of naphthalene, indicative of the production of an EPS matrix, which characterizes some *Variovorax* strains (Jamieson *et al*., 2009). However, none of these strains were capable of developing a structured biofilm around the naphthalene crystal.

The closest relative of the *S. novella* strain N1B is *S. novella* type strain (99% identity of 16S rRNA gene; Kappler *et al*., 2001), which is a strict aerobe incapable of naphthalene degradation and lacking any naphthalene degradation gene within its genome (Kappler *et al*., 2012). In contrast, *S. novella* N1B harbours a naphthalene degradation pathway which is almost identical to the *X. polyaromaticivorans* pathway, characterized by the presence of key dioxygenases with high affinity for oxygen. *X. polyaromaticivorans* was originally described as capable of growth and PAH degradation under extremely low oxygen tensions (below 0.2 ppm; Hirano *et al*., 2004). However, in our microaerophilic enrichments, the *S. novella* strain N1B was able to totally outcompete *X. polyaromaticivorans*, probably after having horizontally acquired its *dbd* pathway. Thus, it seems that the capacity of the *S. novella* strain N1B to develop a structured biofilm in close proximity to the naphthalene crystal is a key factor that provides a strong advantage to the strain for growth with naphthalene under these oxygen‐limiting conditions. In fact, it has been hypothesized from simulated biofilm competition experiments that polymer producers are evolutionary favoured at the expenses of non‐producers (Xavier and Foster, 2007). The ecological benefit driving this selection in our enrichment may be to provide a better competition for the substrate by locating the biofilm producers at the optimum distance from the carbon source to allow efficient mass transfer, simultaneously maintaining a sufficient distance to avoid a possible toxic effect of the naphthalene. The stable presence of *V. paradoxus* in the biofilm suggests that its EPS production capacity favours its presence in the community, probably contributing to the structure of the biofilm structure.

## Concluding remarks

Mixed bacterial biofilms are increasingly being considered as attractive tools in bioremediation protocols for their increased stability and resistance (Demeter *et al*., 2015). In fact, bacteria in the sessile state which form part of biofilms are more resistant to pollutants (Khoei *et al*., 2016) and more effective in pollutant degradation (Edwards and Kjellerup, 2013) than as free‐living cells. In this study, the initial sample collected at the oil–water interface had a medium‐to‐low phylogenetic diversity, numerically dominated by *Betaproteobacteria*, which represented more than 60% of the community. Enrichment under microaerophilic conditions resembling those naturally found in real environmental‐polluted sites produced a drastic change in the community, pointing to oxygen limitation as the main factor driving the structure of the community, selecting for strains that were undetectable in the starting material. Community members bearing degradation pathway enzymes with high affinity for oxygen were selected, whilst additionally the capacity to produce EPS and to develop biofilms was crucial for the final selection. Interestingly, this enrichment evidenced the function of biofilm structure to favour gene transfer, thus generating a naphthalene degrading strain able to grow at very low oxygen tensions and with a striking capacity to develop a physical structure to locate the cells at the appropriate distance from the toxic carbon source. Non‐polar substances such as hydrocarbons can accumulate in the EPS matrix (Späth *et al*., 1998), which would help increase the local concentration of the substrate in proximity to the biofilm producers and biofilm‐associated strains. Finally, no growth was observed when oxygen was completely omitted from the cultures and nitrate was the only terminal electron acceptor (anoxic enrichments), which supports the general view that NRB are actually adapted to microaerophilic conditions, where the different reactions involved in hydrocarbon degradation could be spatial and/or temporally separated in the bacterial communities (Yagi *et al*., 2010).

## Experimental procedures

### Sampling and site description

The groundwater sample used in this study was collected in sterile bottles at 25 m depth from a hydrocarbon contaminated aquifer located under an oil refinery (REPSOL) in Valle de Escombreras (Murcia, Spain) (37°34′20.6″N 0°55′24.7″W). The bottles were sealed under nitrogen gas and stored at 4°C until used. The aerobic and anaerobic enrichment culture of this work started from the biofilm present at the interface between the aquifer water and the hydrocarbon layer (Fig. S1).

### Most probable number enumeration of bacteria

Aerobic and nitrate‐reducing PAH‐degrading bacterial populations were enumerated using the MPN technique. The aerobic and the anaerobic media composition were similar and were prepared as described previously (Martirani‐Von Abercron *et al*., 2016). The medium (9 ml) was distributed in 12‐ml vials (LABCO Limited, Lampeter, Wales, UK) where previously naphthalene, 2‐methylnaphthalene, naphthoic acid and anthracene had been added to the tubes from stock diethyl ether solutions to reach a final concentration of 0.025‰, 0.028‰, 0.035‰ and 0.035‰, respectively, and the solvent had been evaporated before dispensing the medium. Acetate was used as a positive control to a final concentration of 5 mM, and controls with no added carbon source were included. For each sample, triplicate tubes were inoculated with 1 ml of the corresponding dilution in 10‐fold serial dilutions. For the NRB enumeration, the minimal medium containing 5 mM sodium nitrate as the electron acceptor was purged with sterile nitrogen gas and 9 ml was poured into oxygen‐free tubes. Cultures were incubated at 28°C in the dark, and anaerobic growth of NRB was checked during 6 months as the nitrite produced from nitrate respiration as described (Martirani‐Von Abercron *et al*., 2016). Aerobic cultures were incubated with slow shaking (100 rpm) during 1 month, and growth was determined as turbidity. The data are the result of three replicates per sample within the 95% confidence interval.

### Culture conditions, enrichment and isolation procedures

For the cultivation of nitrate‐reducing bacteria (NRB) under strict anoxic conditions, 25 ml of sample was transferred to 120‐ml serum bottles containing 75 ml of modified non‐reduced Widdel mineral medium. Medium preparation and composition were as described previously (Martirani‐Von Abercron *et al*., 2016). Naphthalene (NAP) and 2‐methylnaphthalene (2MN) prepared in 2‐ml sterile anoxic HMN (20 g l^−1^) as carrier were added in each serum bottle. A control with only HMN was included. The cultures were prepared under nitrogen atmosphere and sealed with Teflon‐lined 1‐cm‐thick stoppers. All culture manipulations were carried out under nitrogen atmosphere. To determine growth, the cultures were monitored periodically for nitrite produced from nitrate respiration or for nitrate consumption by ion chromatography as described (Martirani‐Von Abercron *et al*., 2016). Transfers of NRB cultures were made every 3 months by inoculating 10% (v/v) of the cultures into fresh medium. This operation was repeated three times, and after an additional two‐month incubation, samples were taken for pyrosequencing. For the microaerophilic enrichments, the medium composition and the preparation were the same as for the anaerobic one, except that naphthalene provided as crystals was used as the sole carbon source and the rubber stoppers were completely wrapped in a Teflon tape. Teflon tape is slightly permeable to air and thus allows a slow rate oxygen flow through into the culture vessels (see below). In the absence of an inoculum, oxygen only reached saturation after 5–7 days (Fig. S4B). A similar oxygen evolution rate could be obtained by inserting a 30‐gauge needle through the rubber stopper instead of wrapping it with Teflon tape (not shown). Microaerophilic enrichment cultures were incubated at 28°C without shaking. Every month 10% (v/v) of the cultures were inoculated into fresh medium. Samples for pyrosequencing analysis were taken after five (Micro5‐N) and fifteen (Micro15‐N) transfers. Aerobic enrichments were set up starting from the fifth transfer of the microaerophilic culture. Briefly, 5 ml of the fifth microaerophilic enrichment was inoculated in 250‐ml shake flasks containing 50 ml of fresh Widdel minimal medium with naphthalene crystals as the sole carbon source. Flasks were incubated in an orbital shaker at 100 rpm and at 28°C. Transfers (10%) were made every 15 days. Total DNA for pyrosequencing analysis was isolated after the fifteenth (Aer15‐N) and nineteenth (Aer19‐N) transfer. Isolation of aerobic strains was performed at 28°C in Widdel mineral medium supplemented with either naphthalene crystals on the Petri dish cover, which was wrapped with Teflon tape, or 10% Luria–Bertani medium (LB). Isolates were selected by colony morphology and restreaked until pure cultures were obtained. Aerobic growth of the isolates on naphthalene was carried out at 28°C on Widdel mineral medium supplemented with naphthalene crystals.

### Measurement of oxygen concentration in the culture bottles

The set‐up used to measure the oxygen concentration inside the bottles is shown in Fig. S4A. Temperature‐compensated oxygen concentration of the medium was continuously recorded using oxygen‐sensitive optodes (5‐mm‐diameter spots; OXSP5, Pyro Science GmbH, Aachen, Germany) glued to the inner wall of the bottle and connected to a Firesting Optical Oxygen Meter via fibre‐optic cables (SPFIB‐BARE, Pyro Science. K., Aachen, Germany) fixed outside the wall in front of the sensor spot with the aid of a Basic Spot Adapter (SPADBAS). The fibre was connected to a fibre‐optic oxygen meter (Fire Sting O2, Pyro Science GmbH, Aachen, Germany) linked to a computer with Pyro Oxygen Logger software. Incubations were carried out at 28°C. A bottle filled with water and fitted with a temperature sensor was placed in the same chamber for temperature record. The temperature‐compensated dissolved oxygen tension was recorded continuously and expressed as percentage of air saturation. Figure S4B shows the oxygen evolution in sterile and inoculated culture bottles equipped with an oxygen‐sensitive optode placed at the bottom of the bottle wall.

The set‐up used to measure the oxygen concentration inside the bottles is shown in Fig. S4A. Temperature‐compensated oxygen concentration of the medium was continuously recorded using oxygen‐sensitive optodes (5‐mm‐diameter spots; OXSP5, Pyro Science GmbH, Aachen, Germany) glued to the inner wall of the bottle and connected to a Firesting Optical Oxygen Meter via fibre‐optic cables (SPFIB‐BARE, Pyro Science. K., Aachen, Germany) fixed outside the wall in front of the sensor spot with the aid of a Basic Spot Adapter (SPADBAS). The fibre was connected to a fibre‐optic oxygen meter (Fire Sting O2, Pyro Science GmbH, Aachen, Germany) linked to a computer with Pyro Oxygen Logger software. Incubations were carried out at 28°C. A bottle filled with water and fitted with a temperature sensor was placed in the same chamber for temperature record. The temperature‐compensated dissolved oxygen tension was recorded continuously and expressed as percentage of air saturation. Figure S4B shows the oxygen evolution in sterile and inoculated culture bottles equipped with an oxygen‐sensitive optode placed at the bottom of the bottle wall.

### Synthetic microbial community

Strains were first picked from colony into Widdel mineral medium supplemented with 1/10 LB. Late‐exponential‐phase cells were harvested and washed twice with fresh medium without any carbon source. Cell densities were determined based on OD_600_ readings. Co‐culture growth was initiated by inoculating 0.02 OD_660_ units of each strain in N_2_ flushed Widdel mineral medium supplemented with a naphthalene crystal as the sole carbon source. Serum bottles were sealed with Teflon tape‐wrapped rubber stoppers as for the microaerophilic enrichments. Culture was incubated at 28°C without shaking.

### Chemical analysis

For the analysis of nitrate and sulfate, we used duplicate 5 ml aliquots of the original aquifer sample, and quantification was carried out by ion chromatography (IC) using a Metrohm‐761 Compact Ion Chromatograph equipped with a Metrosep A Supp 4–250 column with chemical suppression (50 mM H_2_SO_4_) as described previously (Martirani‐Von Abercron *et al*., 2016). Duplicate samples of the initial contaminated aquifer (5 ml) were used to extract aliphatic and aromatic hydrocarbon compounds, and the deuterated Mix 37 (manufactured by Dr. Ehrenstorfer, Augsburg, Germany) and 5‐alpha‐cholestane (Sigma‐Aldrich, St. Louis, Missouri) were added as internal standards before the extraction. Hydrocarbon analysis was carried out at the Instrumental Technical Services of the Estación Experimental del Zaidín (CSIC), Granada, Spain, by GC‐MS as in Martirani‐Von Abercron *et al*. (2016).

### Total DNA extraction, 16S rRNA gene 454‐pyrosequencing and data analysis

DNA extraction for the initial environmental sample was performed as previously described (Martirani‐Von Abercron *et al*., 2016). For the enrichment cultures and isolated strains, genomic DNA was purified using the Wizard Genomic DNA Purification Kit as recommended by the manufacturer (Promega, Madison, WI, USA). Nucleic acid quantity and purity were determined with a NanoDrop ND‐1000 Spectrophotometer (NanoDrop Technologies Llc, Wilmington, Delaware, USA). A multiplex pyrosequencing amplicon approach was used for the characterization of the bacterial communities. The PCR amplifications of the hypervariable V1–V3 region of the 16S rRNA gene were carried out using the bacterial universal primers 6F and 532R containing 5′ tags with multiplex identifier and sequencing adapters (Table S6). PCR amplifications were performed in 50 μl reactions containing 1× PCR Buffer (Bio‐Rad, Hercules, California), 200 μM dNTPs (Roche, Basel, Switzerland), 0.5 μM of each primer, 1 U of iProof™ High‐Fidelity DNA Polymerase (Bio‐Rad) and 20 ng of target DNA. The PCR program consisted of an initial denaturation step at 98°C for 30 s, followed by 25 cycles at 98°C for 10 s, 50°C for 20 s and 72°C for 30 s, with a final extension at 72°C for 5 min. PCRs were cleaned up using the Qiaquick MiniElute columns (Qiagen, Hilden, Germany) and checked in 1.5% agarose gel. Amplicon products were quantified using Qubit™ fluorometer (Invitrogen, Carlsbad, California), pooled at an equimolar ratio and sequenced using a 454 titanium amplicon sequencing kit and a Genome Sequencer FLX 454 at either Citius (University of Seville) or Macrogen (Korea). Analysis of the 16S 454 pyrosequencing data was performed following the Quantitative Insights Into Microbial Ecology (qiime v. 1.9.0) pipeline (Caporaso *et al*., 2010). Demultiplexing, primer removal, quality‐filtering, chimeras and singletons removal, and alpha‐ and beta‐diversity studies were carried out as defined in Martirani‐Von Abercron *et al*. (2016).

### Full‐length 16S rRNA gene amplification and clone library construction

Near‐full‐length 16S rRNA genes were amplified using bacterial universal primers GM3F and GM4R (Muyzer *et al*., 1995). PCR was performed in 50 μl reactions as previously described (Acosta‐González *et al*., 2013), and the products (~1500‐bp) were cloned in pGEMT (Invitrogen). Positive clones were checked by PCR with vector primers prior to Sanger sequencing. Phylogenetic analysis was performed with the ARB package (Ludwig *et al*., 2004) using the online SINA alignment service and Silva database version SSU Ref 119. OTU's assignation was performed at ≥ 97% sequence similarity. Phylogenetic tree was estimated by the neighbour‐joining method.

### Functional gene amplification

Gene fragments of *bssA*,* ncr*,* nmsA*,* nahA*,* dbd* and *nod* were amplified by PCR using available primer sets and the cycling conditions originally described (Table S6). Genomic DNA from NaphS2 and *Thauera aromatica* K172 strains was used as a positive control for *nmsA* and *ncrA*, and *bssA* amplification, respectively. All PCRs were performed in 50 μl containing 1× PCR Buffer (Bio‐Rad), 200 μM dNTPs (Roche), 0.5 μM of each primer (Sigma‐Aldrich, St. Louis, Missouri, USA), 1 U of iProof™ High‐Fidelity DNA Polymerase (Bio‐Rad), and 10–20 ng of target DNA. The appropriately sized amplicons were purified and cloned in pCR2.1 (TA Cloning Kit; Invitrogen), and 16 positive clones for each gene were Sanger‐sequenced (IPBLN, CSIC, Granada, Spain). In addition to the available primers, six naphthalene dioxygenase (*ndo*) degenerated primers (Table S5) were designed using the CODEHOP (COnsensus‐DEgenerate Hybrid Oligonucleotide Primers) software (http://blocks.fhcrc.org/codehop.html; Rose *et al*., 2003) based on the alignment 29 sequences of dioxygenase proteins of different species (Table S7). A gradient PCR was performed with annealing temperatures ranging from 50°C to 63°C for all primer combinations to determine optimal annealing temperatures. Touchdown PCR was used to avoid amplifying non‐specific sequences: after 1 min of denaturation at 98°C, the first 10 thermal cycles were 10 s at 98°C, 15 s at 63°C to 53°C (the annealing temperature was reduced 1°C per cycle from 63°C to 53°C) and 20 s at 72°C. The remaining 20 cycles were 98°C for 10 s, 55°C for 15 s and 72°C for 20 s, with the last cycle followed by a 5‐min extension at 72°C. Genomic DNA from *P. putida* KT2440 (NAH7; Fernández *et al*., 2012) was used as positive control.

### RT‐PCR assays

Reverse transcriptase PCR (RT‐PCR) of a 200‐bp fragment of *dbdCa* was performed with 75 ng RNA in a final volume of 50 μl using the Titan OneTube RT‐PCR system according to the manufacturer's instructions (Roche Laboratories). Positive (*S. novella* strain N1B DNA) and negative (absence of reverse transcriptase in the assay) controls were included. The primers used were Xdbd and XdbdI (Table S6).

### Biofilm quantification in multiwell plates (crystal violet assay)

Biofilm formation at the air/liquid interface was analysed in LB medium or LB medium saturated with naphthalene under static conditions in polystyrene 96‐well microtiter plates using a modification of the method of Barahona *et al*. (2010). Overnight cultures of each isolate grown in LB medium were diluted in the corresponding medium to an OD_600_ of 0.05. Then, 200 μl of each culture was dispensed in triplicate into the multiwell plates and incubated at 30°C without shaking. Because the biofilm formation kinetics varied for each strain and condition, biofilms were quantified at different time points for 50 h as follows: the cell suspension was removed, the wells were washed with 200 μl of sterile water, and the same volume of crystal violet solution (0.4%, w/v) was added to each well and incubated for 20 min to allow staining of adhered cells. Excess stain was eliminated by rinsing with water. Plates were air‐dried, and 200 μl of 95% ethanol (v/v) was added to each well to extract the crystal violet from cells. Distaining was performed overnight with shaking (40 rpm) after which the DO_540_ was measured on a microplate reader. The values when biofilm formation was highest were selected for each strain.

### Scanning electron microscopy (SEM)

Specimen preparation and SEM were performed at the Scientific Instrumentation Centre (CIC) of the University of Granada. Biofilm samples from microaerophilic cultures growing on naphthalene were fixed for 2 h at 4°C in 2.5% glutaraldehyde prepared in cacodylate buffer, pH 7.4. The fixed samples were rinsed three times (15 min each) with the same buffer at 4°C and incubated for 1 h with 1% osmium tetroxide at room temperature, rinsed three times (5 min) with water and dehydrated in an increasing ethanol concentration gradient from 50% to 100%. Samples were further desiccated with carbon dioxide in a Leica EM CPD300 critical point dryer according to Anderson (1951). Samples were carbon coated in an EMTECH K975X evaporator and examined in a Zeiss SUPRA40VP scanning electron microscope equipped with a Schottky type emission gun.

### Nucleotide sequence accession numbers

The 454 pyrosequencing raw reads have been deposited in the NCBI short‐reads archive database (accession number SRR5417958 to SRR5417967). The partial *dbdB* gene sequences obtained from the clone libraries have been deposited in GenBank under the provisional submission ID 2034219 and 2034262. The 16S rRNA gene clone library sequences have been deposited in GenBank under accession numbers MF156538 to MF156549.

## Conflict of interest

The authors declare that the research was conducted in the absence of any commercial or financial relationships that could be construed as a potential conflict of interest.

## Supporting information


**Table S1.** Aromatic hydrocarbon composition and abundance in the contaminated aquifer initial samples.
**Table S2.** Characterization of the initial polluted aquifer sample.
**Table S3.** Most probable number counts of nitrate reducing and aerobic bacteria in the initial contaminated aquifer sample.
**Table S4.** List of OTUs retrieved from the initial sample and from the different enrichment cultures.
**Table S5.** Functional gene primers used in this study.
**Table S6.** 16S rRNA primers 6F and 532R targeting V1‐V3 region containing 5′ tags with multiplex identifier (MID) and sequencing adapters used for pyrosequencing analysis.
**Table S7.** Naphthalene dioxygenase sequences used to design the CODEHOP primers used in this study.
**Fig. S1.** Aquifer sample showing a visible biofilm growing at the interface between the oil and the water layers.
**Fig. S2.** Aliphatic hydrocarbon distribution (%) in the initial contaminated aquifer sample.
**Fig. S3.** Rarefaction curves for 16S rRNA genes of microbial communities from the initial sample and enrichment cultures.
**Fig. S4.** Oxygen concentration in the microaerophilic culture bottles used in this study. Initially, 50‐100 ml serum bottles filled‐in with medium were flushed with nitrogen gas until oxygen concentration was zero and were then sealed with 1 cm‐thick butyl stopper wrapped up with Teflon tape. An oxygen sensor spot glued at the bottom and connected to a Firesting Optical Oxygen Meter via fibre‐optic cables (Pyro Science. K., Aachen, Germany) allowed continuous record of oxygen concentration. A) Scheme of the bottle and oxygen meter set‐up. B) Oxygen concentration evolution at the bottle bottom: blue and green lines: examples of two non‐inoculated controls; red line, inoculated culture.
**Fig. S5.** Neighbour‐joining tree of the 16S rRNA gene of the bacterial strains isolated in this study and their closest relatives in the databases.
**Fig. S6.** *Xanthobacteraceae* phylogenetic tree using the 16S rRNA V1‐V3 region of the isolates and pyrosequencing reads in the microaerophilic, aerobic and synthetic enrichments.
**Fig. S7.** Phylogeny of the alpha subunit oxygenase component of hydroxylating naphthalene dioxygenase (upper panel) and gentisate 1,2‐dioxygenase (lower panel) partial amino acid sequence retrieved from Subculture 5 and different isolates obtained in this study.
**Fig. S8.** RT‐PCR analysis of total RNA extracted from subculture 15 (+).
**Fig. S9.** Biofilm formation at the air/liquid interface by different isolates in LB (light blue) or LB supplemented with a naphthalene crystal (dark blue) .
