# Supplementary material for: Naphthalene biodegradation under oxygen‐limiting conditions: community dynamics and the relevance of biofilm‐forming capacity
Source: Microb Biotechnol. 2017 Aug 25;10(6):1781–96. doi: 10.1111/1751-7915.12842 (PMC5658598; doi:10.1111/1751-7915.12842)
Supplement: Supplementary file 1 — Table S1. Aromatic hydrocarbon composition and abundance in the contaminated aquifer initial samples. Table S2. Characterization of the initial polluted aquifer sample. Table S3. Most probable number counts of nitrate reducing and aerobic bacteria in the initial contaminated aquifer sample. Table S4. List of OTUs retrieved from the initial sample and from the different enrichment cultures. Table S5. Functional gene primers used in this study. Table S6. 16S rRNA primers 6F and 532R targeting V1‐V3 region containing 5′ tags with multiplex identifier (MID) and sequencing adapters used for pyrosequencing analysis. Table S7. Naphthalene dioxygenase sequences used to design the CODEHOP primers used in this study. Fig. S1. Aquifer sample showing a visible biofilm growing at the interface between the oil and the water layers. Fig. S2. Aliphatic hydrocarbon distribution (%) in the initial contaminated aquifer sample. Fig. S3. Rarefaction curves for 16S rRNA genes of microbial communities from the initial sample and enrichment cultures. Fig. S4. Oxygen concentration in the microaerophilic culture bottles used in this study. Initially, 50‐100 ml serum bottles filled‐in with medium were flushed with nitrogen gas until oxygen concentration was zero and were then sealed with 1 cm‐thick butyl stopper wrapped up with Teflon tape. An oxygen sensor spot glued at the bottom and connected to a Firesting Optical Oxygen Meter via fibre‐optic cables (Pyro Science. K., Aachen, Germany) allowed continuous record of oxygen concentration. A) Scheme of the bottle and oxygen meter set‐up. B) Oxygen concentration evolution at the bottle bottom: blue and green lines: examples of two non‐inoculated controls; red line, inoculated culture. Fig. S5. Neighbour‐joining tree of the 16S rRNA gene of the bacterial strains isolated in this study and their closest relatives in the databases. Fig. S6. Xanthobacteraceae phylogenetic tree using the 16S rRNA V1‐V3 region of the isolates and pyrosequencing reads [file MBT2-10-1781-s001.docx]

***Supporting Information for:***

**Naphthalene biodegradation under oxygen limiting conditions: community dynamics and the relevance of biofilm-forming capacity**

Sophie-Marie Martirani-Von Abercron, Patricia Marín, Marta Solsona-Ferraz, Mayra Alejandra Castañeda-Cataña and Silvia Marqués^*^

Estación Experimental del Zaidín, Department of Environmental Protection, Consejo Superior de Investigaciones Científicas, Granada, Spain.

*Author for correspondence: Silvia Marqués, Estación Experimental del Zaidín, CSIC, C/. Profesor Albareda nº1, E-18008 Granada, Spain, [silvia@eez.csic.es](mailto:silvia@eez.csic.es)

This document includes 6 supplementary tables and 9 supplementary figures.

**Table S1.** Aromatic hydrocarbon composition and abundance in the contaminated aquifer initial samples.

| **Compound** | **ppm (µg/g)** |
| --- | --- |
| Naphthalene | 0.1377±0.082 |
| 1-methylnaphthalene | 0.1868±0.081 |
| 2-methylnaphthalene | 0.2002±0.119 |
| 1,4,5-trimethylnaphthalene | nd |
| 1,4,6,7-tetramethylnaphthalene | 0.0145±0.009 |
| Dibenzothiophene | 0.0299±0.026 |
| Phenanthrene | 0.0244±0.015 |
| Anthracene | 0.0245±0.005 |
| Benzo[a]anthracene | nd |
| Pyrene | 0.0066±0.0007 |
| C2-naphthalene | 0.0294±0.018 |
| Fluoranthene | nd |
| Chrysene | nd |
| **Total** | 0.654 |

nd: not detected.

**Table S2.** Characterization of the initial polluted aquifer sample. The detailed hydrocarbon composition of the sample can be found in supplementary Table S1 and Figure S2.

| **Sample Name** | **Description** | **Location** | **Coordinates** | **Nitrate**  **(μM)** | **Sulphate**  **(μM)** | **Total hydrocarbons**  **(μg/kg)** | |
| --- | --- | --- | --- | --- | --- | --- | --- |
|  |  |  |  |  |  | Aliphatic | Aromatics |
| **INI** | Aquifer below an oil refinery, 25 m depth | Valle de Escombreras, Murcia (Spain) | 37°34'20.6"N 0°55'24.7"W | 84.64±0.24 | 19.27±0.72 | 2130.80 | 654.00 |

**Table S3.** Most probable number counts of nitrate reducing and aerobic bacteria in the initial contaminated aquifer sample. Carbon source are naphthalene (NAP), 2-methylnaphthalene (2MN), 2-naphtoic acid (2NA), anthracene (ANT), acetate (Ace) and no added carbon source (na).

| **Sample name** | **C source** | **MPN counts** | | **95% C.I.** | |
| --- | --- | --- | --- | --- | --- |
| **Anoxic** | **na** | 3,79E+00 | 9,00E-01 | | 1,27E+01 |
|  | **NAP** | 2,86E+00 | 6,30E-01 | | 1,05E+01 |
|  | **2MN** | 7,28E+00 | 1,71E+00 | | 2,12E+01 |
|  | **2NA** | 4,27E+00 | 1,03E+00 | | 1,39E+01 |
|  | **ANT** | 3,79E+00 | 9,00E-01 | | 1,27E+01 |
|  | **Ace** | 9,33E+04 | 2,07E+04 | | 2,71E+05 |
| **Aerobic** | **na** | 9,30E+00 | 2,07E+00 | | 2,71E+01 |
|  | **NAP** | 4,27E+02 | 1,03E+02 | | 1,38E+03 |
|  | **2MN** | 2,40E+04 | 4,76E+03 | | 9,65E+04 |
|  | **2NA** | 1,47E+03 | 2,78E+02 | | 6,32E+03 |
|  | **ANT** | 1,14E+03 | 2,37E+02 | | 3,56E+03 |
|  | **Ace** | 1,38E+07 | 2,68E+06 | | 6,32E+07 |

**Table S4:** see TABLE_S4.xlsx file.

**Table S5.** Functional gene primers used in this study.

| **Target gene** | **Primer set** | **5′-3′ sequence** | **Reference** |
| --- | --- | --- | --- |
| *bssA* | 7772f  8546r | GACATGACCGACGCSATYCT  TCGTCGTCRTTGCCCCAYTT | (Winderl et al., 2007) |
| *bssA, nmsA* | 7768f  8543r | CAAYGATTTAACCRACGCCAT  TCGTCRTTGCCCCAYTTNGG | (von Netzer et al., 2013) |
| *nmsA* | 7363f  7374f  8543r | TCGCCGAGAATTTCGAYTTG  TTCGAYTTGAGCGACAGCGT  TCGTCRTTGCCCCAYTTNGG | (von Netzer et al., 2013) |
| *ncr* | Ncr1f  Ncr1r | CGTTATWCKCCYTGCCGTG  CGATAAGCCATRCADATRGG | (Morris et al., 2014) |
|  | Ncr2f  Ncr2r | TGGACAAAYAAAMGYACVGAT  GATTCCGGCTTTTTTCCAAVT | (Morris et al., 2014) |
| *nahA* | pPAH-F  pPAH-NR700 | GGYAAYGCNAAAGAATTCGTNTGYWSHTAYCAYGGITGGG  CCAGAATTCNGTNGTRTTHGCATCRATSGGRTKCCA | (Hedlund et al., 1999) |
|  | DP1Rieske_f  ARHD2R | TGYMGNCAYMGNGG  AANTKYTCNGCNGSNRMYTTCCA | (Iwai et al., 2011) |
| *ndo* | NAPH-1F  NAPH-1R | TGGCTTTTCYTSACBCATG  DGRCATSTCTTTTTCBAC | (Gomes et al., 2007) |
| *ndo* | NAH-306F | AAGGGCTTCGTGTGCAACTAYCAYGGNTG | This study |
|  | NAH-621F | CGTGGGCGACGCCTAYCAYRTNGG |  |
|  | NAH-627F | GACGCCTACCACGTGGGNTGGRMNCA |  |
|  | NAH-612R | TGGTAGGCGTCGCCCRYRAARTTYTC |  |
|  | NAH-633R | GGCGTGGGTCCAGCCNAYRTGRTA |  |
|  | NAH-1110R | TGTTCATCTGGGTCACGGTCNHCATRTTNTC |  |
| *dbdB* | dbdB-F | GAGGGCCACATATGTCATTCGCG | (Hirano et al., 2007) |
|  | dbdB-R | CCGAAGCGGGTCGACTTTCAC |  |
| *dbdAB* | XdbA_up | GTAGAGAAGGAGAAGGCCAT | This study |
|  | Dbd_R_280 | GTCGTGATCCGCGATTGTCC |  |
| *dbdCa* | XdbdIII | GGACCGCGGCAATCAGGAGTGA |  |
|  | XdbdI | GCCGGACTCTTCGGCGTAGG |  |
| *dbdCaCbCc* | Xdbd | ATGGTCCGCACCTTCTCGGC |  |
|  | XdbdI-Rev | CCGGCGATCGAGGCGTTGTA |  |
| *dbdDE* | XdbdII | AGGCGATCGTCGAGCGGTTC |  |
|  | XdbdII-Rev | CAGCAGCATGCGCCGATGAC |  |
| *dbdCa* | XdbdIII | GGACCGCGGCAATCAGGAGTGA |  |
|  | XdbdI | GCCGGACTCTTCGGCGTAGG |  |
| *nod* | nod684Fv2 | GGCTTSGCRATCCAGTAGAAG | (Zhu et al., 2017) |
|  | nod1706Rv2 | TGYMGNCAYMGNGG |  |

**Table S6.** 16S rRNA primers 6F and 532R targeting V1-V3 region containing 5′ tags with multiplex identifier (MID) and sequencing adapters used for pyrosequencing analysis.

| **Sample name** | **Primer name** | **Adaptor** | **Key** | **MID** | **Primer** | **Primer position** |
| --- | --- | --- | --- | --- | --- | --- |
| **Ia** | M1A | CGTATCGCCTCCCTCGCGCCA | TCAG | ACGAGTGCGT | TCAGAGTTTGATCCTGGCTCAG | 6F |
|  | M1B | CTATGCGCCTTGCCAGCCCGC | TCAG | ACGAGTGCGT | CACCGCGGCKGCTGGCAC | 532R |
| **Ib** | M2A | CGTATCGCCTCCCTCGCGCCA | TCAG | ACGCTCGACA | TCAGAGTTTGATCCTGGCTCAG | 6F |
|  | M2B | CTATGCGCCTTGCCAGCCCGC | TCAG | ACGCTCGACA | CACCGCGGCKGCTGGCAC | 532R |
| **Anox-N** | 11A | CGTATCGCCTCCCTCGCGCCA | TCAG | TGATACGTCT | TCAGAGTTTGATCCTGGCTCAG | 6F |
|  | 11B | CTATGCGCCTTGCCAGCCCGC | TCAG | TGATACGTCT | CACCGCGGCKGCTGGCAC | 532R |
| **Anox-2MN** | 84A | CGTATCGCCTCCCTCGCGCCA | TCAG | CGAGACACTAT | TCAGAGTTTGATCCTGGCTCAG | 6F |
|  | 84B | CTATGCGCCTTGCCAGCCCGC | TCAG | CGAGACACTAT | CACCGCGGCKGCTGGCAC | 532R |
| **Anox-HMN** | M2A | CGTATCGCCTCCCTCGCGCCA | TCAG | ACGCTCGACA | TCAGAGTTTGATCCTGGCTCAG | 6F |
|  | M2B | CTATGCGCCTTGCCAGCCCGC | TCAG | ACGCTCGACA | CACCGCGGCKGCTGGCAC | 532R |
| **Micro5-N** | 86A | CGTATCGCCTCCCTCGCGCCA | TCAG | CGAGTCATCGT | TCAGAGTTTGATCCTGGCTCAG | 6F |
|  | 86B | CTATGCGCCTTGCCAGCCCGC | TCAG | CGAGTCATCGT | CACCGCGGCKGCTGGCAC | 532R |
| **Micro15-N** | 92A | CGTATCGCCTCCCTCGCGCCA | TCAG | CGTAGCTCTCT | TCAGAGTTTGATCCTGGCTCAG | 6F |
|  | 92B | CTATGCGCCTTGCCAGCCCGC | TCAG | CGTAGCTCTCT | CACCGCGGCKGCTGGCAC | 532R |
| **Micro12s-N** | M4A | CGTATCGCCTCCCTCGCGCCA | TCAG | AGCACTGTAG | TCAGAGTTTGATCCTGGCTCAG | 6F |
|  | M4B | CTATGCGCCTTGCCAGCCCGC | TCAG | AGCACTGTAG | CACCGCGGCKGCTGGCAC | 532R |
| **Aer15-N** | 91A | CGTATCGCCTCCCTCGCGCCA | TCAG | CGTACAGATAT | TCAGAGTTTGATCCTGGCTCAG | 6F |
|  | 91B | CTATGCGCCTTGCCAGCCCGC | TCAG | CGTACAGATAT | CACCGCGGCKGCTGGCAC | 532R |
| **Aer19-N** | 87A | CGTATCGCCTCCCTCGCGCCA | TCAG | CGATCGTATAT | TCAGAGTTTGATCCTGGCTCAG | 6F |
|  | 87B | CTATGCGCCTTGCCAGCCCGC | TCAG | CGATCGTATAT | CACCGCGGCKGCTGGCAC | 532R |

**Table S7.** Naphthalene dioxygenase sequences used to design the CODEHOP primers used in this study.

| *Strain* | Accession number |
| --- | --- |
| *Pseudomonas fluorescens* | AAL07262 |
| *Pseudomonas putida* | WP_011117400 |
| *Burkholderia* sp. | ACT53249 |
| *Pseudomonas stutzeri* | AAD02136 |
| *Paraburkholderia* *sartisoli* | AAD09872 |
| *Marinomonas* *profundimaris* | WP_024024133 |
| *Nevskia* *ramosa* | WP_022978279 |
| *Polaromonas* *naphthalenivorans* CJ2 | AAZ93388 |
| *Alteromonas* *naphthalenivorans* | WP_013785992 |
| *Diaphorobacter* sp. DS2 | AGH09226 |
| *Comamonas* *testosterone* | AAF72976 |
| *Delftia* sp. Cs1-4 | WP_013801305 |
| *Acidovorax* sp. JS42 | AAB40383 |
| *Acidovorax* sp. NA3 | ACG70971 |
| *Sphingomonas* sp. VKM B-2434 | AHF58581 |
| bacterium enrichment pahAc3 | AFH77961 |
| *Paraburkholderia* *fungorum* | AGN90996 |
| *Novosphingobium* *aromaticivorans* | WP_011906634 |
| *Cycloclasticus* *zancles* | WP_016389425 |
| *Cycloclasticus* sp. NY93E | ADD10619 |
| *Sphingomonas* *polyaromaticivorans* | PhnA 2CKF_A |
| *Novosphingobium* sp. PP1Y | WP_013834067 |
| *Sphingomonas* sp. | A4 BAD34447 |
| *Comamonas* sp. MQ | AEV91670 |
| *Pseudomonas chlororaphis* | ADM26645 |
| *Burkholderia* *cepacia* | AAL50021 |
| *Variovorax* *paradoxus* | WP_041942906. |


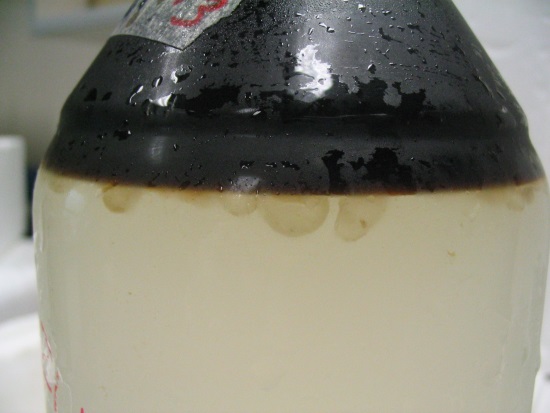


**oil**

water

biofilm

**Figure S1.** Aquifer sample showing a visible biofilm growing at the interface between the oil and the water layers.

**Figure S2.** Aliphatic hydrocarbon distribution (%) in the initial contaminated aquifer sample. The total amount of aliphatic hydrocarbons in the sample was 0.654 µg/ml**.**


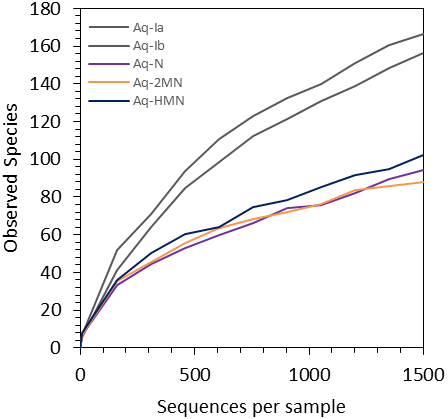


INIa

INIb

Anox-NAP

Anox-2MN

Anox-HMN


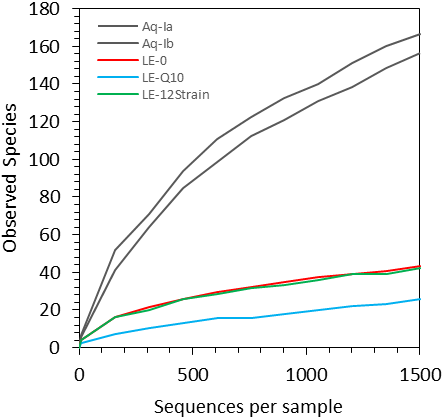


INIa

INIb

Micro5-N

Micro15-N

Micro-12s-N


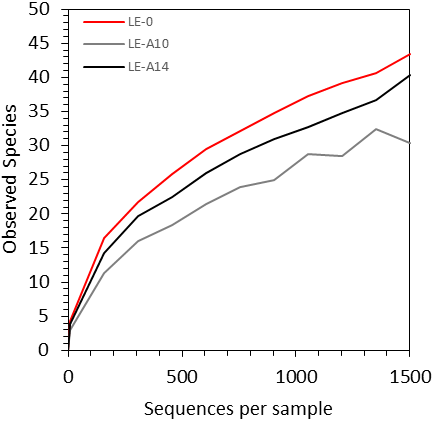


Micro5-N

Aer15-N

Aer19-N

**Figure S3.** Rarefaction curves for 16S rRNA genes of microbial communities from the initial sample and enrichment cultures. Upper panel, anoxic enrichments; Middle panel, microaerophilic enrichments; Lower panel, aerobic enrichments. Labels are as in Table 2. Clones were grouped into phylotypes at a level of ≥97% sequence similarity.


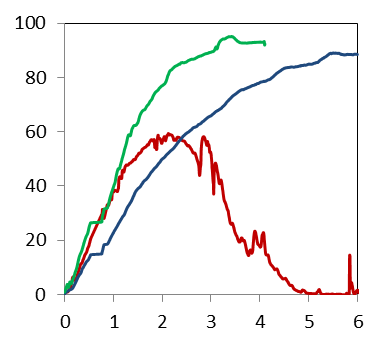


**Time (days)**

**% oxygen saturation**

O_2_meter


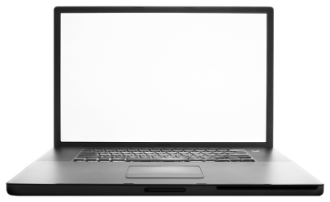

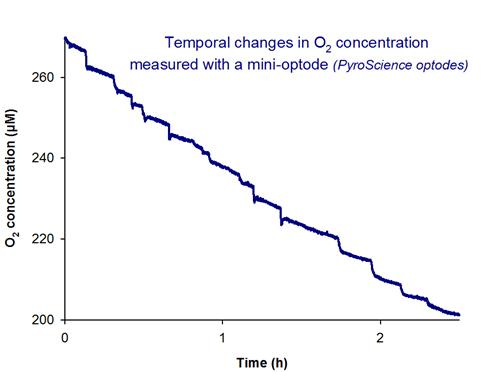


**B**

**A**

O_2_sensor spot

**Figure S4.** Oxygen concentration in the microaerophilic culture bottles used in this study. Initially, 50-100 ml serum bottles filled-in with medium were flushed with nitrogen gas until oxygen concentration was zero and were then sealed with 1 cm-thick butyl stopper wrapped up with Teflon tape. An oxygen sensor spot glued at the bottom and connected to a Firesting Optical Oxygen Meter via fibre-optic cables (Pyro Science. K., Aachen, Germany) allowed continuous record of oxygen concentration. A) Scheme of the bottle and oxygen meter set-up. B) Oxygen concentration evolution at the bottle bottom: blue and green lines: examples of two non-inoculated controls; red line, inoculated culture.

**958**

**968**

denovo31221_B2.LE.A10_27361_f__Comamonadaceaeg__Variovorax

denovo30278_B4.LE.Q10_1376_f__Comamonadaceaeg__Variovorax__paradoxus

**HQ005420.1_*Variovorax_paradoxus*_strain_HB44**

denovo9575_B5.LE.12STRAIN_59053_f__Xanthomonadaceaeg__Pseudoxanthomonass__

denovo6311_AQ1_29514_f__Xanthomonadaceaeg__Pseudoxanthomonass__

denovo25802_AQ1_42044_f__Xanthomonadaceaeg__Pseudoxanthomonass__

**962**

**CP003093.2:3414512-3416060_*Pseudoxanthomonas_spadix*_BD-a59_complete_genome**

denovo14227_B1.LE.0_106423

**972**

**NR_115989.1_*Epilithonimonas_lactis*_strain_H1**

**KT766029.1_*Chryseobacterium*_sp._ERMR1:04**

denovo14536_B2.LE.A10_21993_f__Pseudomonadaceaeg__Pseudomonas

**KF863235.1_*Pseudomonas_stutzeri*_strain_N2**

**943**

denovo9091_AQ2MN_19000_f__Pseudomonadaceaeg__Pseudomonas

**963**

**LC007966.1_*Pseudomonas_denitrificans*_isolate:_05CF15-5C**

**NR_028706.1_*Pseudomonas_veronii*_strain_CIP_104663**

**KF975414.1_*Brevundimonas_vesicularis*_strain_KK6**

**AJ244706.1_*Brevundimonas_mediterranea*_strain_V4.BO.18**

denovo716_B5.LE.12STRAIN_1942_f__Caulobacteraceaeg__s__

**951**

denovo31963_AQ2MN_3643f__Rhizobiaceaeg__s__

**960**

**NR_044216.1_*Rhizobium*_*selenitireducens*_strain_B1**

**NR_074219.1_*Starkeya_novella*_strain_DSM_506**

**964**

denovo24185_B1.LE.0_26733_f__Xanthobacteraceaeg__s__

denovo11305_B1.LE.0_6427_f__Xanthobacteraceae

FR733686.1_*Aquabacter_spiritensis*_type_strain_DSM9035T

**NR_112205.1_*Xanthobacter_polyaromaticivorans*_strain_127W**

denovo27209_B5.LE.12STRAIN_23404_f__Xanthobacteraceaeg__Xanthobacters__autotrophicus

denovo670_B1.LE.0_327_f__Xanthobacteraceae

denovo10294_B1.LE.0_19862_f__Xanthobacteraceae

**966**

**952**

**983**

denovo18658_B5.LE.12STRAIN_148598_f__Microbacteriaceaeg__Microbacteriums__maritypicum

**LN890176.1_*Microbacterium_oxydans*_strain_M90**

KX369591.1_*Microbacterium_oxydans*_strain_AE038-20

JN881579.1_*Candidatus_Caldiarchaeum_subterraneum*_clone_PNG_TBR_A55

0,05

***Variovarax***

***Pseudoxanthomonas***

***Epilithonimonas***

***Pseudomonas***

***Brevundimonas***

***Rhizobium***

***Starkeya***

***Xanthobacter***

***Microbacterium***

**Figure S5.** Neighbour-joining tree of the 16S rRNA gene of the bacterial strains isolated in this study and their closest relatives in the databases. Representative closest QIIME OTUs for each strain (labelled as “denovo”) are included. The bar represents 0.03 substitutions per site.

HQ882805.1_Xanthobacter_sp._W30

**NR_112205.1_Xanthobacter_polyaromaticivorans_strain_127W**

denovo31548_Aer19-N_196203_f__Xanthobacteraceaeg__Xanthobacters__autotrophicus

denovo17083_12Strains_20917_f__Xanthobacteraceaeg__Xanthobacters__autotrophicus

denovo4695_Micro5-N_48060_f__Xanthobacteraceaeg__Xanthobacters__autotrophicus

denovo670_Micro5-N_327_f__Xanthobacteraceae

denovo10294_Micro5-N_19862_f__Xanthobacteraceae

denovo11279_Aer15-N_131361_f__Xanthobacteraceae

denovo24662_Micro5-N_32754_f__Xanthobacteraceae

denovo30474_12Strains_308895_f__Xanthobacteraceaeg__Xanthobacters__autotrophicus

denovo36483_Micro5-N_176601_f__Xanthobacteraceaeg__Xanthobacters__autotrophicus

denovo29250_Aer15-N_97553_f__Xanthobacteraceae

denovo22704_Micro5-N_300608___Xanthobacteraceaeg__Xanthobacters__autotrophicus

denovo16626_Micro5-N_165653_f__Xanthobacteraceae

denovo19162_12Strains_234137_f__Xanthobacteraceaeg__Xanthobacters__autotrophicus

denovo12408_12Strains_3605_f__Xanthobacteraceae

denovo18445_Aer19-N_183899_f__Xanthobacteraceaeg__Xanthobacters__autotrophicus

denovo23078_Aer19-N_52381_f__Xanthobacteraceaeg__Xanthobacters__autotrophicus

denovo24967_Micro5-N_207913_f__Xanthobacteraceae

NR_074255.1_Xanthobacter_autotrophicus_strain_Py2

**FR733686.1_Aquabacter_spiritensis_type_strain_DSM9035T**

NR_104747.1_Aquabacter_spiritensis_strain_SPL-1

AB245351.1_Xanthobacteraceae_bacterium_Gsoil_062

denovo5725_Aer19-N_187763_f__Xanthobacteraceae

JQ659763.1_Starkeya_novella_strain_R5-394-1

JQ659901.1_Starkeya_sp._R7-573

denovo11305_Micro5-N_6427_f__Xanthobacteraceae

denovo24185_Micro5-N_26733_f__Xanthobacteraceaeg__s__

denovo9070_Aer19-N_28427_f__Xanthobacteraceae

**NR_074219.1_Starkeya_novella_strain_DSM_506**

JQ659760.1_Starkeya_novella_strain_R5-393

denovo17311_12Strains_63640_f__Xanthobacteraceaeg__s__

denovo32578_Micro5-N_94666_f__Xanthobacteraceae

denovo34290_Micro5-N_152404_f__Xanthobacteraceae

NR_026153.1_Thauera_aromatica__train_K172

0.05

**Clon 966**

**Clon 964**

**Figure S6.** *Xanthobacteraceae* phylogenetic tree using the 16S rRNA V1-V3 region of the isolates and pyrosequencing reads in the microaerophilic, aerobic and synthetic enrichments. Sequences labelled with “denovo” are the pyrosequencing read OTUs retrieved from each dataset, as they are classified by QIIME. The closest type strains are highlighted in bold.


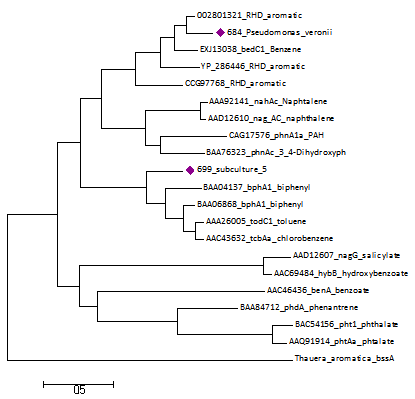


***nahA***

002801321_RHD_aromatic

EXJ13038_*bedC1*_Benzene

AAA92141_*nahAc*_Naphthalene

YP_286446_RHD_aromatic

CCG97768_RHD_aromatic_uncultured

CAG17576_*phnA*1_PAH

AAD12610_*nagAc*_Naphthalene

BAA76323_*phnAc*_3,4-dihydroxyphe

BAA04137_*bphA1*_biphenyl

BAA06868_*bphA1*_biphenyl

AAA26005_*todC1*_toluene

AAC43632_*tcbAa*_chlorobenzene

AAD12607_*nagG*_salicylate

AAC69484_*hybB*_hydroxybenzoate

AAC46436_*benA_*benzoate

BAA84712_*phdA_*phenantrene

BAC54156_*pht1_*phthalate

AAQ91914_*phtAa_*phthalate

*Thauera aromatica_bssA*

◆ **Clon 963 *Pseudomonas veronii***

◆ **Subculture 5**

***dbdB*** *(X. poliaromaticivorans)*


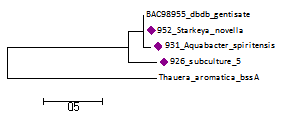


BAC98955_dbdB_*X.polyaromaticivorans*

◆ **Clon 964 *S. novella***

◆ **Clon 966 *X. polyaromaticivorans***

◆ **Subculture 5**

*Thauera aromatica_bssA*

**Figure S7.** Phylogeny of the alpha subunit oxygenase component of hydroxylating naphthalene dioxygenase (upper panel) and gentisate 1,2‑dioxygenase (lower panel) partial amino acid sequence retrieved from Subculture 5 and different isolates obtained in this study. The trees were rooted with unrelated *bssA* gene product as out-group.


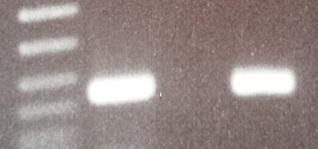


320

242

190

**M + c D**

**Figure S8.** RT-PCR analysis of total RNA extracted from subculture 15 (+). A negative control (c) with RNA where the reverse transcription step had been omitted and a positive control with DNA (D) from *S. novella* strain N1B were included. M, molecular weight marker.


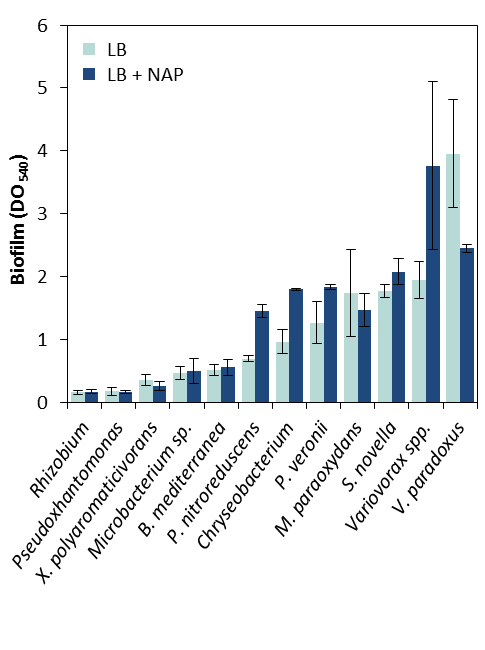


**Figure S9.** Biofilm formation at the air/liquid interface by different isolates in LB (light blue) or LB supplemented with a naphthalene crystal (dark blue). Assays were carried out in triplicate in microtiter plates as described in the Experimental Procedures section.

**References**

Gomes, N.C.M., Borges, L.R., Paranhos, R., Pinto, F.N., Krögerrecklenfort, E., Mendonça-Hagler, L.C.S., and Smalla, K. (2007) Diversity of ndo Genes in Mangrove Sediments Exposed to Different Sources of Polycyclic Aromatic Hydrocarbon Pollution, *Applied and Environmental Microbiology* **73**: 7392-7399.

Hedlund, B.P., Geiselbrecht, A.D., Bair, T.J., and Staley, J.T. (1999) Polycyclic Aromatic Hydrocarbon Degradation by a New Marine Bacterium, Neptunomonas naphthovorans gen. nov., sp. nov, *Applied and Environmental Microbiology* **65**: 251-259.

Hirano, S., Morikawa, M., Takano, K., Imanaka, T., and Kanaya, S. (2007) Gentisate 1,2-dioxygenase from Xanthobacter polyaromaticivorans 127W, *Biosci Biotechnol Biochem* **71**: 192-199.

Iwai, S., Johnson, T.A., Chai, B., Hashsham, S.A., and Tiedje, J.M. (2011) Comparison of the specificities and efficacies of primers for aromatic dioxygenase gene analysis of environmental samples, *Appl Environ Microbiol* **77**: 3551-3557.

Morris, B.E., Gissibl, A., Kummel, S., Richnow, H.H., and Boll, M. (2014) A PCR-based assay for the detection of anaerobic naphthalene degradation, *FEMS microbiology letters* **354**: 55-59.

von Netzer, F., Pilloni, G., Kleindienst, S., Kruger, M., Knittel, K., Grundger, F., and Lueders, T. (2013) Enhanced gene detection assays for fumarate-adding enzymes allow uncovering of anaerobic hydrocarbon degraders in terrestrial and marine systems, *Appl Environ Microbiol* **79**: 543-552.

Winderl, C., Schaefer, S., and Lueders, T. (2007) Detection of anaerobic toluene and hydrocarbon degraders in contaminated aquifers using benzylsuccinate synthase (bssA) genes as a functional marker, *Environmental microbiology* **9**: 1035-1046.

Zhu, B., Bradford, L., Huang, S., Szalay, A., Leix, C., Weissbach, M., et al. (2017) Unexpected Diversity and High Abundance of Putative Nitric Oxide Dismutase (Nod) Genes in Contaminated Aquifers and Wastewater Treatment Systems, *Appl Environ Microbiol* **83**.
